# Supplementary material for: Intraductal xenografts show lobular carcinoma cells rely on their own extracellular matrix and LOXL1
Source: EMBO Mol Med. 2021 Feb 22;13(3):e13180. doi: 10.15252/emmm.202013180 (PMC7933935; doi:10.15252/emmm.202013180)
Supplement: Supplementary file 1 — Appendix [file EMMM-13-e13180-s001.pdf]

Intraductal xenografts show lobular carcinoma cells rely on their own extracellular matrix and LOXL1

APPENDIX.

Table of contents.

Appendix Figure Legend. Pages 2 – 3

Appendix Figure S1 – Page 4

Appendix Figure S2 – Page 5

Appendix Figure S3 – Page 6

Appendix Figure S4 – Page 7

Appendix Figure S5 – Page 8

Appendix Table S1: Antibodies used in this study - Page 9

Appendix Table S2: Primers used in this study - Page 10

Appendix Table S3: RNA scope probe for LOXL1 used in this study - Page 10

Author List RLS: Gynecologists, oncologists, and pathologists who provided material support - Page 10

Appendix Figure Legend

Fig S1 Transcriptional profiling of ILC and Non-ILC ER+ HER2- patient tumor cells

A Table showing characteristics of primary breast tumors used in this study to generate intraductal PDXs.

B Experimental scheme showing the intraductal injection approach used to generate RNA sequencing data from ILC and non ILC ER+ HER2-PDXs. Breast cancer cells are transduced with RFP-luciferase expressing lentiviruses and injected via the teat into the milk ducts of NSG females. Tumor growth is monitored by bioluminescence imaging.

C Barplot showing the number of protein coding genes which are differentially expressed between ILC and non-ILC xenografts.

D Volcano plot showing differentially expressed genes between ILC and non-ILC patient derived tumor cells, n=3. All highlighted genes have *p*-values < 0.05 according to the limma model used for differential expression analysis. Genes with log2(FC) >0.5 in red and log2FC <0.5 in blue. Names of selected genes are indicated.

Fig S2 Bioinformatic analysis of genes differentially expressed between ILCs and non-ILC ER+ patient tumor cells

A-F RNA Sequencing was performed on ILC and non-ILC tumor cells derived by RFP-based FACS sorting from intraductal PDXs. Differentially expressed genes are ranked based on their p-value and fold change.

A-E GSEA plots showing correlation with genes differentially expressed in lobular carcinoma vs normal ductal breast cells (A), genes down-regulated in HMLE cells (immortalized nontransformed mammary epithelium) after *CDH1* knockdown by RNAi (B), genes involved in cell cycle (left), in G2M checkpoint (middle), and E2F target genes (right) (C), myc targets genes (left), mTOR (middle) and PI3K/AKT /mTOR signaling pathway genes (right) (D) as well as genes involved in interferon-alpha response (left), Androgen signaling response (middle), and TNFA Signaling via NFKB pathway (E).

F Motif activity response analysis associated with EPAS and BCL3 as well as FOXM1 and TBL1XR1 transcription factors as predicted by ISMARA.

Fig S3. TCGA breast tumors gene expression profiles of non-ILC and ILC.

A, B Violin plot showing normalized counts of ILC (n=127) versus non-ILC (n=490) of selected genes associated with matrisome (*ELN*, *COL1A1*, *COL6A1, COL14A1)*. Statistical significance determined by Student's unpaired t-test, two-tailed.

Fig S4. Global gene expression profle of SUM44 BAPN treated intraductal xenografts.

A, B Log10(radiance) curves of tumor growth for the MM134 (left) and SUM44 xenografts (right) treated early (A) and in the metastatic settings (B). For statistical analysis, mixed-effects linear models with spline regression (when applicable) were used. Likelihood-ratio tests were used for model comparison, i.e. to check whether the treatment/group covariate is significant.

C Principal component analysis (PCA) of RNAseq global gene expression data separating samples by treatment.

D Barplot showing the number of differential expressed protein-coding genes between vehicle and BAPN treated xenografts.

E Dotplot of log counts per million (CPM) from individual RNAseq samples (n=3) of selected genes.

F CNET plot of ReactomePA of genes up regulated in BAPN treated xenografts.

G Volcano plot showing genes, which are differentially expressed between vehicle and BAPN-treated SUM44 xenografts; N=3, all highlighted genes have p-values < 0.05 according to the limma model used for differential expression analysis. Genes with log2(FC) >0.5 in red and log2FC <0.5 in blue. Names of selected genes are indicated.

H GSEA plots showing gene sets that are differentially regulated between BAPN and PBS treated mice.

Fig S5. *LOXL1* knockdown and ILC growth.

A, B Log10(radiance) curves of tumor growth for the scramble and sh*LOXL1* MM134 (A) and SUM44 (B) xenografts. For statistical analysis, mixed-effects linear models were used. Likelihood-ratio tests were used for model comparison, i.e. to check whether the treatment/group covariate is significant

Sflomos et al. Appendix Figure S1

A

Tumor Number	Patient age (years)	% ER	% PR	Ki67 index	Subtype
**69**	75	100	100	16	ILC
**73**	69	100	0	26	ILC
**76**	31	100	60	80	non-ILC ER+ HER2-
**78**	78	100	80	10	ILC
**81**	39	98	10	35	non-ILC ER+ HER2-
**99**	57	100	70	20	non-ILC ER+ HER2-

B

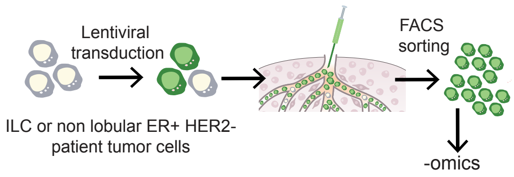

C

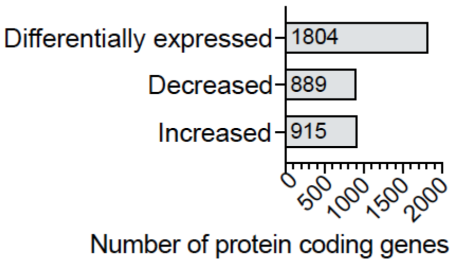

D

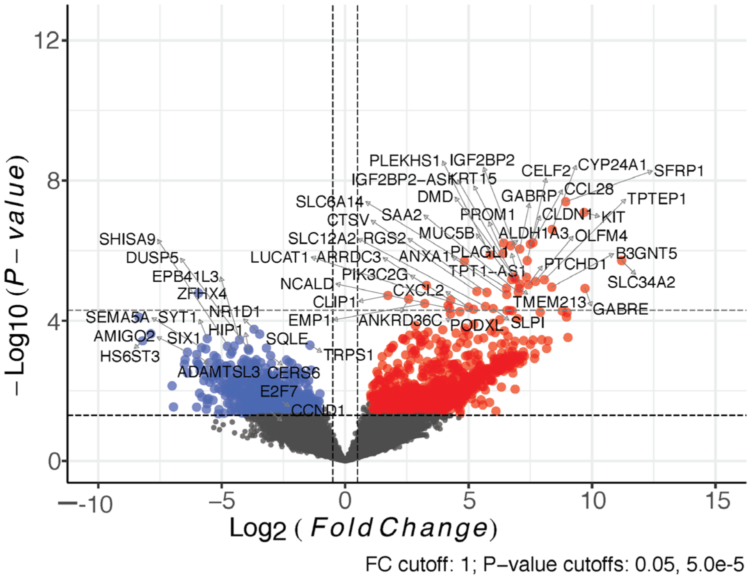

Sflomos et al.

Appendix Figure S2

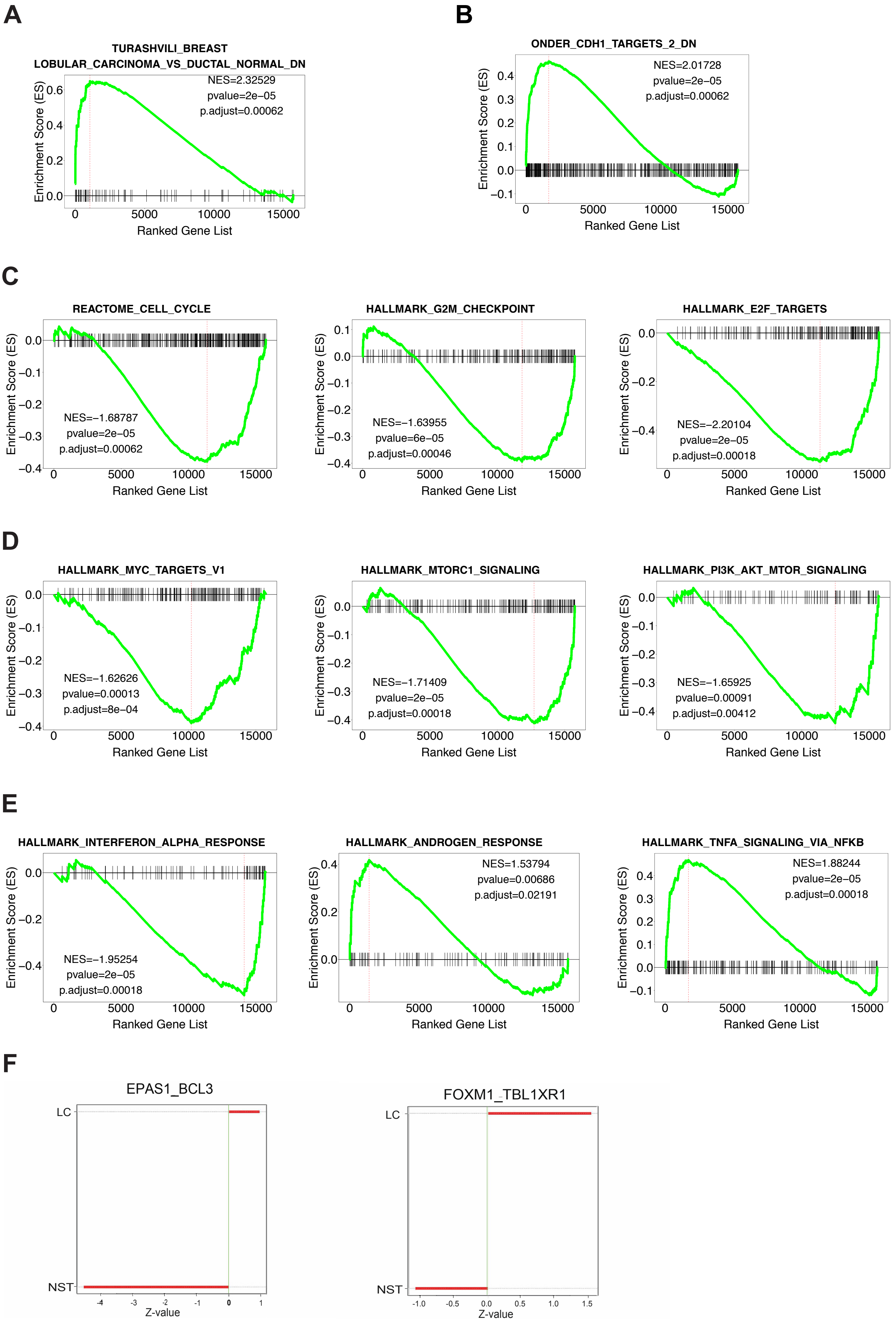

Sflomos et al.,

Appendix Figure S3

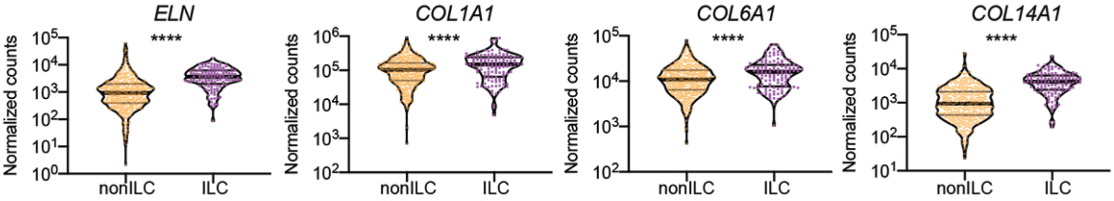

Sflomos et al.   Appendix Fig. S4

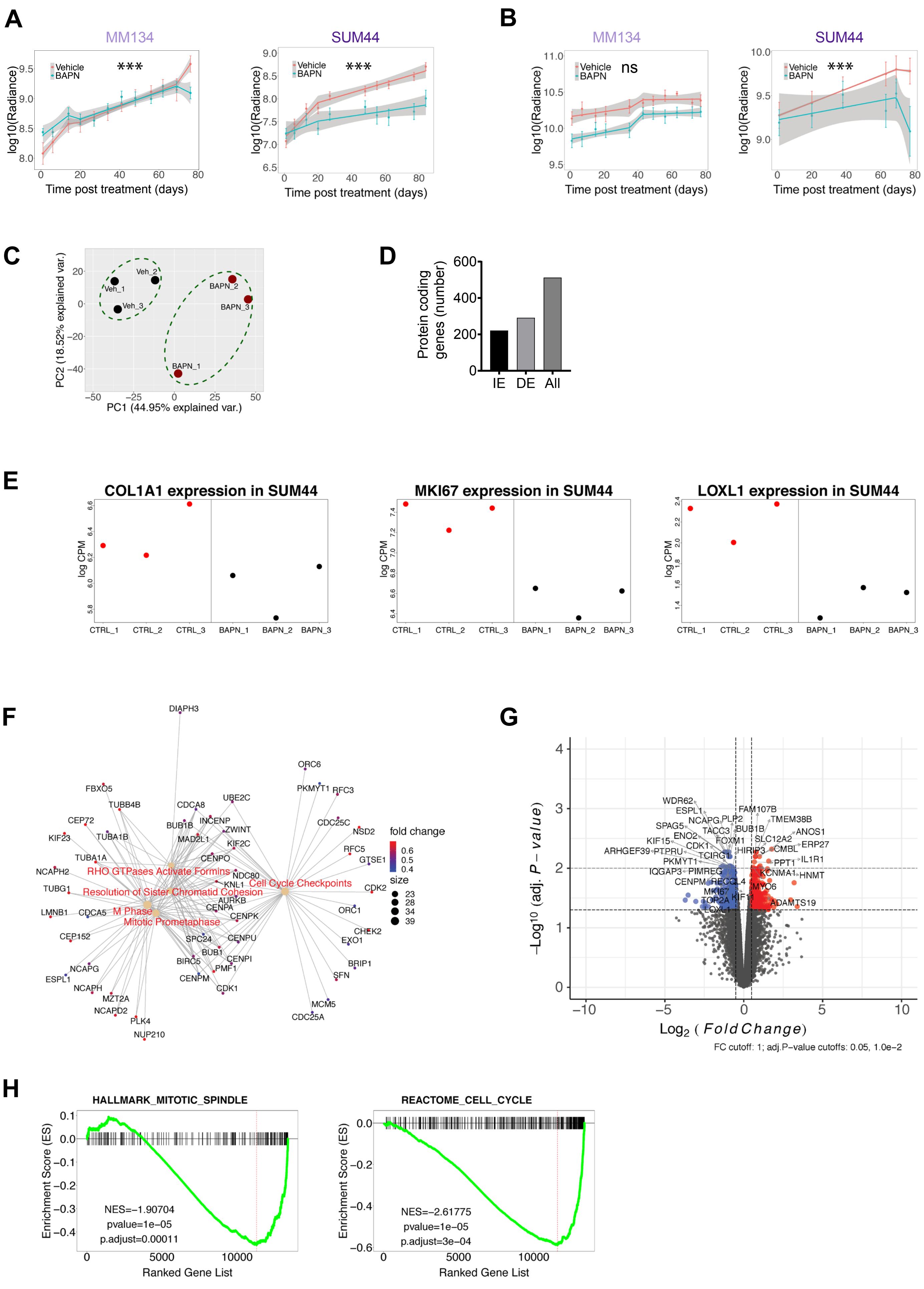

Sflomos et al.

Appendix Fig. S5

A

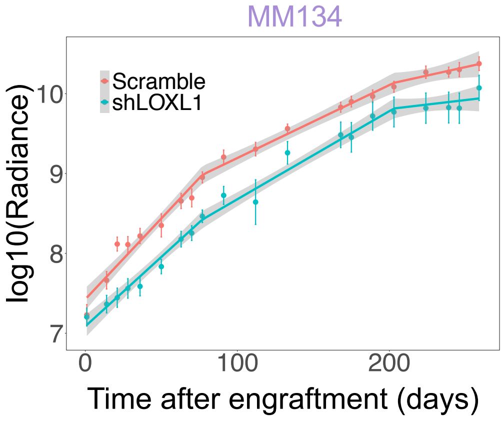

B

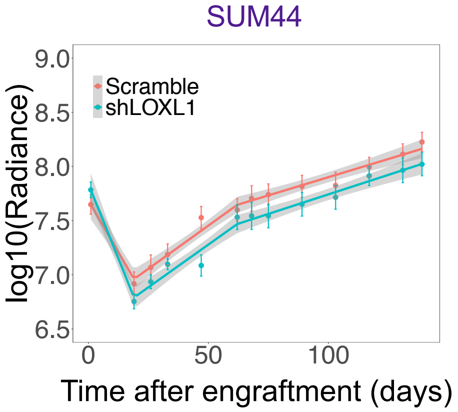

Appendix Table S1: Antibodies used in this study

**Antibody - Target**	**Producer**	**Catalog Number**	**Species**	**Clone**	**Primary dilution**	**Used in Figures**	***Gene***
**Primaries**							
LOXL1	Abcam	ab81488	Rabbit	na	1/200	Fig.4	*https://www.ncbi.nlm.nih.gov/gene/4016*
Ki67	Roche	790-4286	Rabbit	30-9	Ready to use	Fig.2	https://www.ncbi.nlm.nih.gov/gene/4288
ER	Roche	790-4286	Rabbit	SP1	Ready to use	Fig.2	https://www.ncbi.nlm.nih.gov/gene/2099
ER	Zytomed	RBK018	Rabbit	SP1	Ready to use	Fig.2	*https://www.ncbi.nlm.nih.gov/gene/2099*
ER	Santa Cruz	sc-8002	Mouse	F-10	1/200	Fig.4	*https://www.ncbi.nlm.nih.gov/gene/2099*
PR	Roche	790-2223	Rabbit	1E2	Ready to use	Fig.2	https://www.ncbi.nlm.nih.gov/gene/5241
E-cadherin	Cell signaling	sc-8426	Rabbit	G-10	1/400	Fig.2	*https://www.ncbi.nlm.nih.gov/gene/999*
SMA	Thermo Fisher Scientific	RB-9010	Rabbit	na	1/400	Fig.2J	https://www.ncbi.nlm.nih.gov/gene/59
GFP	Santa Cruz	sc-9996	Mouse	B-2	1/500	Fig.2I	*https://www.ncbi.nlm.nih.gov/protein/AAB51347.1?report=genbank&log$=protalign&blast_rank=1&RID=CB0V2C46015*
GFP	Thermo Fisher Scientific	PA5-22688	Rabbit	na	1/400	Fig.6	*https://www.ncbi.nlm.nih.gov/protein/AAB51347.1?report=genbank&log$=protalign&blast_rank=1&RID=CB0V2C46016*
**Secondaries**						Fig.2	
Alexa 568	Thermo Fisher Scientific	A-10042	rabbit	na	1/800	Fig.2	
Alexa 568	Thermo Fisher Scientific	A-10037	mouse	na	1/800	Fig.2	
Alexa 488	Thermo Fisher Scientific	A-21206	rabbit	na	1/800	Fig.2	
Alexa 488	Thermo Fisher Scientific	A-11029	mouse	na	1/800	Fig.2	

Appendix Table S2: Primers used in this study

Primer sequences		
**Gene**	**Forward (5' to 3')**	**Reverse (5' to 3')**
LOXL1	CTG TGC TGC GGA GGA GAA	GTA GTG GCT GAA CTC GTC CA
36B4	CTTCCCACTTGCTGAAAAGG	CGACTCCTCCGACTCTTCCT

Appendix Table S3: RNA scope probe for LOXL1 used in this study

**Probe**	**Cat. Number**	**Link**
Probe- Hs-LOXL1	470751	https://acdbio.com/search/site/%252ALOXL1%252A/cms/probes/webspecies/human

Author List 1 RLS: Gynecologists, oncologists, and pathologists who provided material support

Dr Didier JALLUT, General Director of RLS

Dr Pierre Bohanes

Dr Stéphane Cochet

Dr Marc Gander

Dr Aurélie Sivade

Dr Nam Tran

Dr Pierre-Michel GENOLET

Dr Nehad AKRAM

Dr Pierre KOVALIV

Dr Jean-Paul CHATELAIN

Dr Philippe BRACHER

Dr Catalin STAN

Dr Martine FRANCIOLI

Dr Catherine BECK

Dr Sylvia BONANOMI SCHUMACHER

Dr. Minh-Tri NGUYEN

Dr Ebtisam ALCHAB

Dr Bijan GHAVAMI

Dr Eric CHARDONNENS

Dr Frank GRUTTER

Dr Pu Yan

Dr Monika CHYZYNSKA

Dr Massimo Bongiovanni

Dr Lorenzo TAMINELLI
