# Supplementary figures and images for: Intraductal xenografts show lobular carcinoma cells rely on their own extracellular matrix and LOXL1
Source: EMBO Mol Med. 2021 Feb 22;13(3):e13180. doi: 10.15252/emmm.202013180 (PMC7933935; doi:10.15252/emmm.202013180)

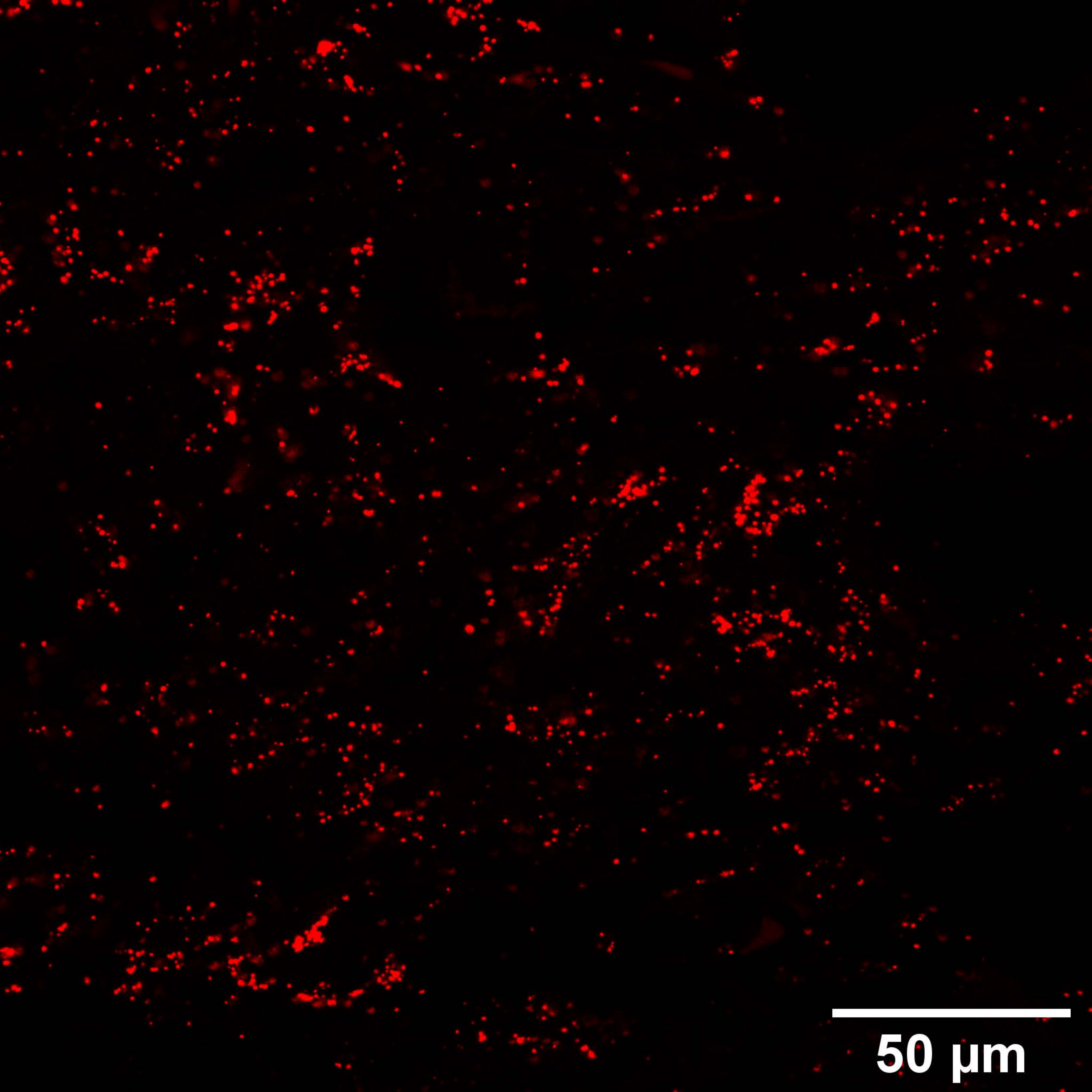

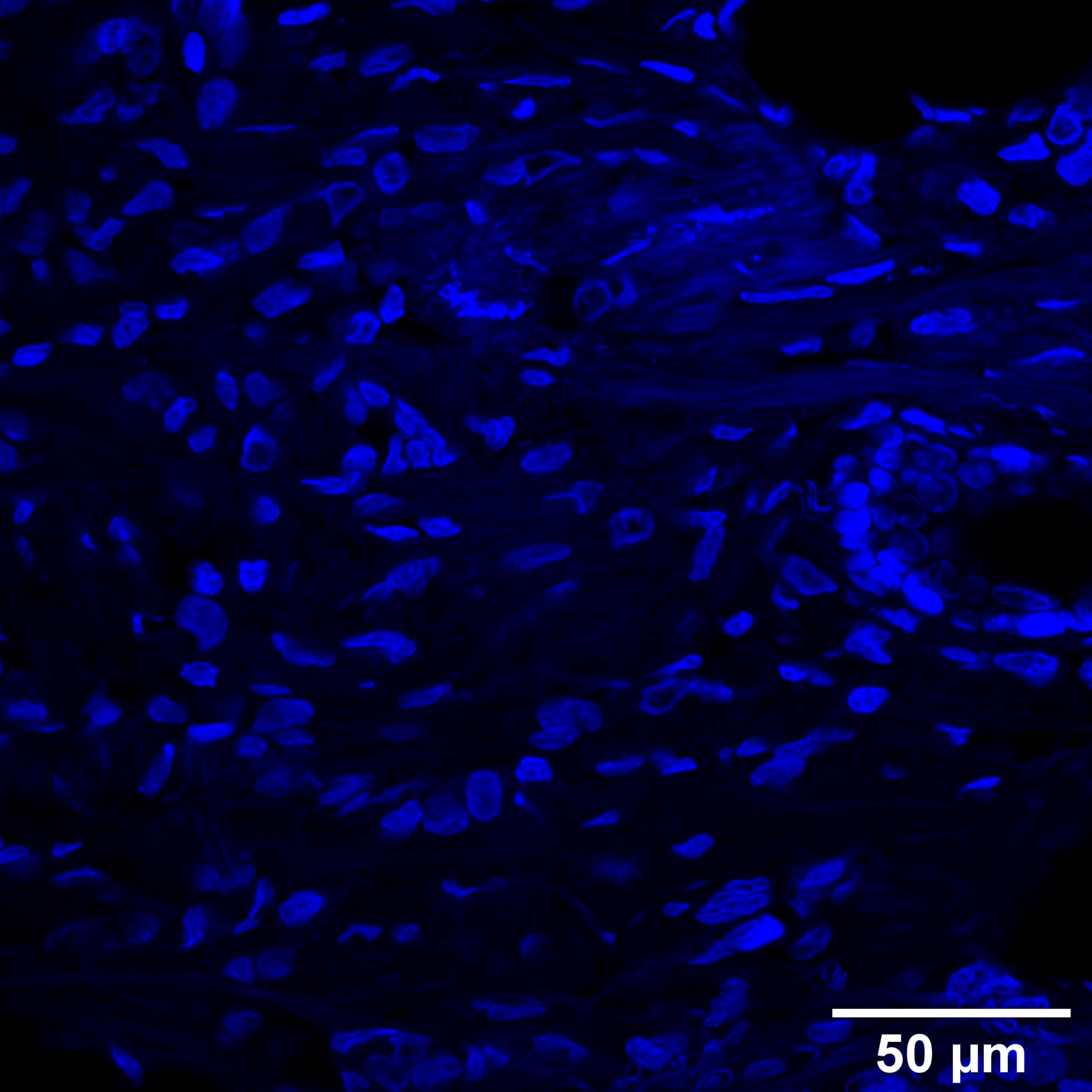

50 μm

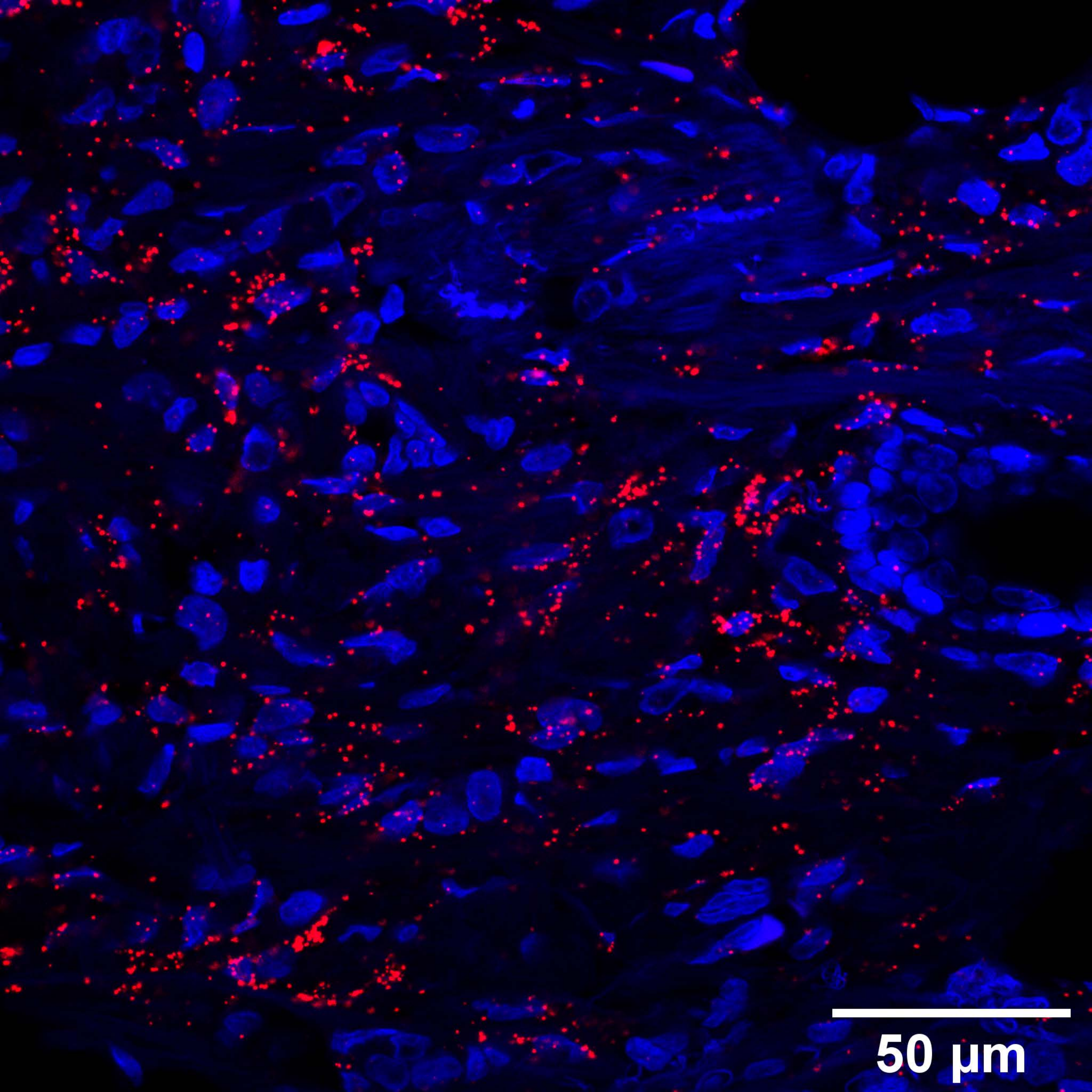

50  $\mu\text{m}$

Supplement: Supplementary file 3 — Source Data for Figure 4 [file EMMM-13-e13180-s002.pdf]
